# Macrophage activation syndrome revealing Hodgkin’s lymphoma: a case report

**DOI:** 10.11604/pamj.2021.38.109.27417

**Published:** 2021-02-03

**Authors:** Mustapha Mechtoune, Rajae Tissir, Ilias Tazi

**Affiliations:** 1 Service d'Hématologie Clinique et de Greffe de Moelle Osseuse, Faculté de Médecine et Pharmacie, Centre Hospitalier Universitaire (CHU) Mohammed VI, Université Cadi Ayyad, Marrakech, Maroc

**Keywords:** Hodgkin lymphoma, macrophage activation syndrome, hemophagocytis, case report

## Abstract

Macrophage activation syndrome (MAS) is a rare immunologic syndrome, rapidly fatal in the absence of specific etiological treatment. It is defined by clinical, biological and cyto-histological criteria. Numerous etiologies have been described in MAS, the association with Hodgkin lymphoma (LH) is exceptional. We report the case of a young woman in whom a macrophage activation syndrome reveals a Hodgkin's lymphoma.

## Introduction

Macrophagic activation syndrome (MAS) is a rare disease characterized by a highly exaggerated and uncontrolled immune response and is potentially fatal if it is not rapidly managed [1]. Diagnosis is dependent on a non-specific combination of clinical and biological symptoms, which requires a cytological or histological investigation of hemophagocytosis and a thorough investigation of the etiology [1]. Macrophagic activation syndrome (MAS) can be primary, familial, related to a disorder of the immune system, especially natural killer (NK) or T-natural killer lymphocytes or secondary and reactive to an underlying pathology. The treatment of the etiology of MAS allows controlling the evolution. Hemopathies responsible for reactive MAS are mainly T or NK lymphomas [2]. The association with LH is exceptional [2]. We report the case of LH revealed by a MAS in a young patient.

## Patient and observation

A 28-year-old woman who, a month before admission, had suffered from asthenia with a weight loss that evolved in a context of altered general condition and fever that was not calculated. On clinical examination, the general condition is very deteriorated (WHO stage 3), fever at 38°C. There was no lymph node hypertrophy on admission and no hepatosplenomegaly on abdominal examination. On the biological level, patient presents a microcytic anemia (hemoglobin: 8.1g/dl), microcytic anemia (mean globular volume (MGV): 78.5 fl), and thrombocytopenia (9000 G/l), leukopenia (leukocytes: 1580 G/l), neutropenia (PNN: 850 G/l), lymphopenia (lymphocyte: 500 G/l), and lymphocytopenia (lymphocyte: 500 G/l). A syndrome of inflammation (sedimentation rate (SV): 49 mm in the first hour, fibrinogen: 5.1 g/l, CRP: 86 mg/l), as well as liver cytolysis (ASAT: 348UI/L , ALAT: 49UI/L , GGT: 96UI/L , Alkaline phosphatases: 158UI/L , total bilirubin: 59 mg/l). Lactate dehydrogenase (LDH) is 1234UI/L, triglycerides 5.72 mmol/l (N: 0.35-1.7) and ferritinemia 2017 g/l (N: 30-400). Infectious balance is negative (blood cultures, serology of atypical germs, PCR of Herpes viridae, Herpes simplex virus 1 (HSV1), Herpes simplex virus 2(HSV2), Parvovirus, varicella zoster virus (VZV), cytomegalovirus (CMV) , Epstein-Barr virus (EBV) , Human Herpesvirus (HHV6) and (HHV8), Sputum stain for Koch bacillus (BK) and gene-expert are negatives, coprocultures and PCR of gastrointestinal tract are negatives).

The myelogram is rich with the presentation of very many images of hemophagocytosis, compatible with MAS. An axillary lymphadenopathy developed one week after the patient's admission, 1 cm x 1 cm in diameter, mobile, solid, painful; removal of the lymphadenopathy is carried out as an emergency procedure. The anatomopathological study revealed a modified and infiltrated architecture of an essentially mononuclear inflammatory element arranged in layers with a few giant cells and fibrosis of the lymph node pulp scattered with rare cells with enlarged nuclei with a prominent nucleolus and a clear cytoplasm that is fairly abundant and poorly limited in favor of scleroderodular LH. The immunohistochemical study found positive anti-CD 30 antibodies, anti MUM1 positive, anti CD15 negative and anti PAX 5 inconclusive antibodies. This result is compatible with LH bone marrow infiltration. The diagnosis of stage IVb scleroderodular LH is retained.

For extension assessment and given the limited resources available in our context for a positron emission tomography (PET) scan, a cervico-thoraco-abdomino-pelvic computed tomography (CT) scan shows mediastinal and left axillary lymph node involvement associated with homogenous hepato-splenomegaly and multiple effusions. Hepatitis B virus (HBV), Hepatitis C virus (HCV) and the human immunodeficiency virus (HIV) viral serology are negative and the echocardiography shows an ejection fraction of 75%. Patient receives 4 courses of dexamethazone 40 mg/day then ABVD (Doxorubicin 25 mg/m^2^/d, Vinblastine 6 mg/m^2^/d, Bleomycin 10 mg/m^2^/d, Dacarbazine 375 mg/m^2^/d on aay 1 and day 15) for 8 courses of treatment with a partial remission then ICE second ligne protocol (Etoposide 100 mg /m^2^/d IV day 1 to day 3, Holoxan 5g/m^2^/d to day 2, Mesna 6g/m^2^ to day 2, Carboplatin formula AUC 5 to day 2 for 4 cures with good evolution.

## Discussion

Diagnosis of MAS is made by a combination of clinical, biological and cytological signs. Diagnostic criteria were recently re-defined [3]. Clinical symptoms are: fever, altered general condition, splenomegaly, icterus, hepatomegaly, lymphadenopathies are frequent [4]. Several biological anomalies are reported during MAS, but are non-specific. It is the association with clinical signs that suggests this diagnosis, bicytopenia and pancytopenia are constant, thrombocytopenia is generally less than 100,000/mm^3^, in this patient we observed a deep pancytopenia. The anemia, normochromic, normocytic, agenerative, is rapid and profound [4]. Hypertriglyceridemia and hyperferritinemia are the most suggestive anomalies in MAS when combined with cytopenias [5], which was the case in this patient. Hypofibrinogenemia is encountered in 35-85% of cases [5]. Clinically, icterus is evidence of hepatic involvement, so the hepatic work-up is always perturbed during MASs. The LDH level is generally high.

It is the myelogram that confirms the diagnosis of MAS. Myelogram shows images of hemophagocytosis which are indispensable for the diagnosis [6]. The positive diagnosis of hemophagocytosis syndrome is difficult and is based on a set of non-specific clinical arguments. The histiocyte society has proposed a set of eight criteria, five of which must be present in order to establish the diagnosis [7]. Generally, the etiologic assessment during MAS is exhaustive [6]. In our observation, the diagnostic difficulties were due to the exceptional association between LH and MAS in the young patient. The major deterioration in general condition made exploration difficult, making the whole etiological assessment negativistic. Recently, cytokine dosage is very useful to confirm the diagnosis, which is not available in our context. Soluble interleukin-2 receptor (sCD25) is one of the diagnostic criteria but this test is not easily performed in routine practice due to limited laboratory resources [8]. Interleukin 2 is a potent modulator of T, B, NK lymphocyte and macrophage activation. Its action is mediated by its specific receptor, sCD25 and its increase is constant during MAS [8]. Interleukin 18 is a pro-inflammatory cytokine of the interleukin 1 family. This mediator is produced by monocytes/macrophages and maintains a Th1-type immune response [9]. During MAS, interleukin 18 is overproduced [10].

The diagnosis of MAS is therefore confirmed in our patient, the exhaustive etiological investigation in the context of the etiological assessment was negative, during her hospitalization in the department, the patient developed an axillary adenopathy allowing the diagnosis of mixed-cellularity LH. In a series of 34 cases of MAS with LH, we note that there was a male predominance [11], reporting the case of a young female patient. The prognosis of the patients is poor when the MAS related to lymphoma compared to the other etiologies, in those MASs with lymphoma the median survival was short (83 days), as was the overall survival (8%), in contrast in MASs with viral infections the survival is close to 83% [11,12]. This means that an appropriate and urgent management is required, as well as the introduction of an immunmodulator treatment, with substitution of all deficient organs before the chemotherapy is started, since the biological perturbations of the hepatic and renal balance in our patient against indicates the start of certain chemotherapy molecules.

MASs are generally associated with T or NK lymphomas, the discovery of LH is an exceptional situation [12]. Seven cases have been described in isolation, up to the national series of 34 patients reported by Ménard *et al*.[11]. LH associated with MAS appears to be a particular entity. All the patients are stage IVb in Ann Arbor's classification, as in this patient's case. An association is noted in 94% of cases with EBV [13]. Finally, the evolution rarely is favorable. In total, the LH forms combined with MAS are distinguished by a presentation similar to the LH appearing in HIV-positive patients. EBV infection raises the hypothesis of an immunodeficiency against EBV because the detection of high levels of antibodies against EBV antigens in patients with LH, therefore patients with a history of infectious mononucleosis are at high risk of developing LH [14]. EBV deoxyribonucleic acid (DNA) was detected in 20-25% of LH biopsies by polymerase chain reaction (PCR) and fluorescence in situ hybridization (FISH) [14]. Our patient has received 8 courses of ABVD (doxorubicin, bleomycin, vinblastine, dacarbazine) [13]. The evaluation report shows a partial response to ABVD and the patient received ICE second-line chemotherapy (ifosfamide, carboplatin and etoposide) with a clinical and biological response and a favorable evolution [15].

## Conclusion

MAS is a rare and grave disease, the mortality rate of which can reach 50% of the cases, diagnosis is based on an association of non-specific clinical and biological symptoms, requiring a haemophagocytosis cytological investigation and a fairly exhaustive etiological investigation, it can be primary or secondary to various affections, in our case, MAS was secondary to a LH.
